# Medical and Philosophical News

**Published:** 1782

**Authors:** 


### SECTION III.

### Medical and Philosophical News.

THE Royal College of Phyficians at Nancy have propofed the following prize queftions: " 1. What are the unwholefome proper-
" ties of fnow water, ice water, or the waters
" of chalky and gypfeous foils? What affinities
" and diftinctions are there between thefe four
" fpecies of water, relative to their chemical
" compofition and their dietetic effects? What
" is the reafon that all waters which contain
" chalk or gypfum, as well as thofe which de-
" rive their fource from melted fnow or ice, are
" not equally unwholefome? Why do the two
" firft produce the fame effects as the two laft of
" thefe waters, notwithftanding the difference in
" many refpects that exifts between them?"

" 2. What degree of influence have they in the
" production of certain popular or endemial dif-
" eafes, particularly in *Bronchocele*, *Scrophula*, and
" *Rachitis?* Does this influence extend to calcu-
" lous and gouty affections? Will this inquiry
" lead to the difcovery of any analogy between
" the lymphatic or glandular fyftem, and the

" bones

" bones and articulations; or to afcertain whe-
" ther the unwholefome effects of thefe different
" waters take place in the procefs of chylifica-
" tion or in that of any other particular fe-
" cretion?"

As it will be difficult for any one to examine all the waters in queftion, Differtations will be received which treat only of one fpecies, and as many medals, of 100 livres each, will be diftributed as there may be found works deferving the prize in the opinion of the committee appointed by the College. The differtations are to be written in French or Latin, and fent to Dr. Harmant, Prefident of the College, before May 1, 1784.

---

The Royal Academy of Surgery at Paris have propofed the following queftion for a prize medal of the value of 500 livres: " What may be
" the influence of the paffions of the mind in
" chirurgical difeafes, and what are the means
" of obviating their effects?" The Differtations on this fubject are to be written in French or Latin, and fent to M. Louis, Secretary to the Academy, before December 31, 1782.

The Royal Medical Society at Paris have offered a premium of 300 livres to the person who shall "determine by chemical analysis the nature of the antiscorbutic remedies procured from the tribe of cruciform plants."—This class *(cruciformes)* in Tournefort's method, corresponds with the *Tetradynamia* of Linnæus. The dissertations are to be written in Latin or French and sent to M. Vicq D'Azyr, Secretary of the Society, before the 1st of May, 1783.

---

Extract of a Letter from a Physician at Gottingen to a Member of the Society. Dated Gottingen April 13, 1782.

"A very ingenious paper on the receptacles of air, in birds, has lately been communicated to our Royal Society, by Professor Merrem, who lectures here on the natural history of the ancients. Among other experiments he made a hole in the os humeri of a pigeon, through which he injected wax, and by this means completely filled all the aerial receptacles. He next injected the arteries of a hen with blue, and the receptacles with red wax. He is convinced that the aspera arteria has no openings between the bronchia, through which the air can pass into the cavity of the thorax, as some have asserted. He observes

ſerves that the lungs are covered with a thin membrane in which there are ſeveral foramina, through which the air paſſes into the different receptacles. This paper, which will probably appear in the next volume of our Commentaries, will be illuſtrated by ſeveral curious engravings.

"I ſhall ſend you by the firſt opportunity that preſents itſelf, ſeveral inaugural Diſſertations, which have lately been printed here. One of them, by Dr. B. F. Munch, relates to *the uſe of the Belladona in Canine Madneſs.* It ſeems that his father, who is a clergyman at Clöze, has for many years adminiſtered the root of this plant in powder with great ſucceſs, and in a great number of caſes. The reſult of this practice is given in the theſis. To very young children he gives at firſt one grain: in adults he begins with ten grains, repeating and gradually increaſing the doſe every other night, it being neceſſary, we are told, to wait forty-eight hours between each doſe. After taking the medicine, the patient is to lie in bed, and promote perſpiration by means of warm diluting drink. If it occaſions a troubleſome vertigo, which, as ſometimes has been the caſe, does not eaſily go off, an emetic, or purge, or a few ſpoonfuls of vinegar, generally remove it. As a preventative, three doſes are ſaid to be, in general, ſufficient.

ſufficient. If the ſymptoms of hydrophobia are actually preſent, the ſame remedy is preſcribed in a large doſe, with the addition of warm bathing, clyſters, anointing the wound with oil, &c. I muſt candidly confeſs to you, that I am far from being ſanguine in my expectations from this new remedy, notwithſtanding the many fine things that Dr. Munch ſays of it; but as ſo little has hitherto been done in theſe melancholy caſes, it certainly has a claim to our attention."

---

We have lately ſeen the proſpectus of a new work, which is to be entitled "Encyclopedie "Methodique ou par ordre des Matieres, par "une Societé des Gens de Lettres, de Savans et "d'Artiſtes, precédé d'un vocabulaire univerſel "ſervant de Table pour tout l'ouvrage, orné des "Portraits de M. M. Diderot et d'Alembert, "premiers editeurs de l'Encyclopedie." It is to be printed in two forms; one in 4to. with three columns, in 42 volumes of letter-preſs, and ſeven volumes of plates; the other in 8vo. with two columns, in 84 volumes, beſides ſeven of plates. The price to ſubſcribers will be 672 livres; to non-ſubſcribers, 798; and the whole work is to be finiſhed in five years. None of the volumes are to be had ſeparate.

This

This work is to conſiſt of twenty-ſix dictionaries, of which we ſhall juſt enumerate the titles, with the names of the authors, that our readers may form ſome idea of the magnitude of the undertaking, and of the manner in which it is likely to be executed.—1. Mathematics, 2 vols. 4to. by Abbé Boſſut and M. de la Lande; 2. Natural Philoſophy, 1 vol. by M. Mongez; 3. Phyſic, 2 or 3 vols. by M. Vicq D'Azyr; 4. Anatomy and Phyſiology, human and comparative, 1 vol. by the ſame, aſſiſted, in the comparative part, by M. D'Aubenton; 5. Surgery, 1 vol. by M. Louis; 6. Chemiſtry, Metallurgy, and Pharmacy, 2 vols. by Meſſieurs de Morveau, du Hamel, and Moret; 7. Agriculture, 2 vols. by Abbé Teſſier, M. Thourin, and M. Fougeroux de Bondaroy; 8. The Natural Hiſtory of Animals, 3 vols. by Meſſieurs D'Aubenton, Mauduyt, and Guenau de Montbeillard; 9. Botany, 2 vols. by the Chevalier de la Marck; 10. The Natural Hiſtory of Foſſils, 1 vol. by M. D'Aubenton; 11. Natural Hiſtory, and Phyſical Geography, 1 vol. by M. Deſmarets; 12. Geography, ancient and modern, 2 vols. by Meſſieurs Robert, Maſſon de Morvilliers, Mentelle, and Bonne; 13. Antiquities, 1 vol. by M. Court de Geblin; 14. Hiſtory, 2 vols. by M. Gaillard;

Gaillard; 15. Theology, 2 vols. by Abbé Bergier; 16. Philofophy, ancient and modern, 1 vol. by M. Naiglon; 17. Metaphyfics, Logic, and Morality, 1 vol. by M. Guenau de Montbeillard; 18. Grammar and Literature, 1 vol. by Meffieurs Marmontel and Beauzée; 19. Civil Law, 3 vols. by Abbé Remy; 20. Finances, 1 vol. by M. Digeon; 21. Political Oeconomy, 1 vol. by Abbé Beaudeau; 22. Commerce, 1 vol. by Abbé Beaudeau and M. Benoit; 23. Marine, 2 vols. by Meffieurs Vial de Clairbois and Blondeau, Profeffors at Breft; 24. Art of War, 2 vols. by Meffieurs de Keralio and de Pommereuil; 25. Fine Arts, 1 vol. by Abbé Arnaud and M. Suard; 26. Arts and Trades, 4 vols. by Meffieurs Roland de la Platiere, Perier, Fougeroux de Bondaroy, Defmarets, and others; 27. General Index to the whole, 1 vol. 4to.

---

It has been remarked of Epidemic Catarrhs, that they have ufually been the moft widely and generally fpreading epidemic known. This obfervation is particularly applicable to the late, or rather prefent difeafe of this kind, for its influence can hardly yet be faid to be at an end.

We have been informed by a very refpectable

Phyfi-

Phyſician, who is lately returned to this country from Ruſſia, that the Influenza began to prevail at St. Peterſburgh about the beginning of February. It was traced from Tobolſky, and was ſuppoſed to have come thither from China; ſo that, like the generality of former epidemics of this kind, it ſeems to have been of Aſiatic origin. It ſoon ſpread through the whole Ruſſian empire, appearing with nearly the ſame ſymptoms as it has done here, but with more of the inflammatory type, a circumſtance which may be aſcribed to the difference of ſeaſon and climate. Many perſons, at St. Peterſburgh, had a ſecond, and even a third attack. Theſe relapſes were generally attended with conſiderable inflammatory ſymptoms, and oftentimes proved fatal.

This diſeaſe ſpread rapidly through all the Northern parts of Europe, and about the beginning of May began to be felt in London. The ſpring here, as well as in other parts of Europe, had been unuſually backward; and the weather, for ſome weeks previous to the appearance of the epidemic, had been cold and wet. The wind had blown chiefly from the N. and N. E. quarters.

About the 20th of May it became extremely general, and continued to be ſo during the remainder of the month. Some hot days in the beginning of June ſeemed to have diſarmed it of much of its violence; and after the ſecond week we heard but of few freſh attacks. In Kent and Suſſex, and in the Weſtern counties, it began to be felt nearly about the ſame time as in London; but in the Northern parts of the kingdom it appeared ſomewhat later. At Edinburgh, tho' as general, it was much milder than in London. One of our correſpondents, who reſides in Kent, near the coaſt, informs us, that it paſſed off very lightly in that part. "Few (ſays he) have eſcaped it; but I know not of a ſingle inſtance of danger."

We have been aſſured, by a very ingenious phyſician, that an entire family in the city eſcaped, altho' all their neighbours were attacked with it; and ſeveral inſtances have fallen within our own obſervation of one or more perſons of a family eſcaping while the reſt were affected.

The ſymptoms produced by this epidemic were extremely various. In the greater number of patients it began with a ſtoppage at the noſe, laſſitude, pains in the back and limbs, a ſenſe of weight and a dull heavy pain about the forehead, chiefly in the direction of the frontal ſinuſes, and extending from thence to the temples and ſometimes to one or both ears. On the ſecond or third day, in ſome caſes ſooner, in others later, a copious diſcharge of mucus began to take place from the noſe, and the patients were troubled with a frequent dry cough, the ſeat of which ſeemed to be principally about the larynx. While theſe ſymptoms were going on, and eſpecially during the firſt days of the diſeaſe, the pulſe was quickened to 100, and in many to 120 ſtrokes in a minute, but without any increaſed fulneſs.

Some had violent pains about the face with little or no cough; others had a troubleſome ophthalmia with a diſcharge of acrid tears. In ſeveral the night fever was attended with ſlight delirium. The patients who were ſo affected were generally weak and extremely irritable. In ſome the diſcharge from the noſe preceded the pain in the head, while in others it did not come on till two or three days after it, and we met with ſome caſes where there was a violent throbbing pain of the head without any diſcharge from the noſtrils. In ſome it produced a ſlight ſore throat, and ſeveral complained that they loſt their taſte for three or four days after the violence of the ſymptoms was abated.—Of all this variety of ſymptoms the pain about the

ſinuſes

ſinuſes of the face ſeemed to be the moſt conſtant.

Some who had it ſlightly got well in three or four days, while others were extremely ill with it for a fortnight, and we ſtill every day meet with patients whoſe complaints of cough, loſs of fleſh, &c. are owing to the ſeverity of the Influenza, or to its having been improperly treated.

As hardly any valetudinarians eſcaped it, and as in theſe it generally appeared with the greateſt ſeverity, ſo in general it was attended with dangerous ſymptoms only in patients of this claſs. We have heard of ſeveral inſtances where it ſeemed to have haſtened the death of the patient by coming on in the advanced ſtage of phthiſis or other dangerous complaints; but we have ſeen no caſe where it proved fatal to patients who were previouſly in a good ſtate of health.

Of the medicines that were preſcribed in this complaint a ſmall doſe or two of emetic tartar given at the beginning ſo as to excite nauſea and a ſlight diaphoreſis, ſeemed to have the beſt effects. The oily mixture, with nitre, and *Elixir Paregoricum*, were uſeful in mitigating the cough. In many caſes the inhaler was uſed with advantage. By theſe ſimple means, and attention to diet, the diſorder, in the worſt caſes, was eaſily removed.

removed. Of the many patients we faw in this way, there were but few who feemed to require bleeding, and thefe were chiefly pregnant women, in whom there is always a difpofition to inflammatory diathefis.

We have feen no inftance of a fecond attack, that is, no cafe where the difeafe occurred again with the fame or any thing like the fame fymptoms as at firft; but we have met with feveral patients, chiefly females, who fome days after their recovery from a fevere attack of the Influenza have had a troublefome deep feated cough and flight fever, which by proper care have generally gone off in a few days.

---

Dr. Brouffonnet, of Montpellier, a very ingenious naturalift who is at prefent in London, is preparing a natural hiftory of fifhes, which he intends to publifh in numbers. Each number is to confift of ten plates and five fheets of letter-prefs in Latin.

---

M. Horne, one of the phyficians to the Duke d'Orleans, has begun to publifh at Paris a new periodical work, entitled, *Journal de Medecine Militaire*, a number of which is to appear every third third month. It confifts of obfervations made in military hofpitals.

---

M. Willemet, apothecary at Nancy, in the *Journal de Medecine* for February 1782, has given the natural and medical hiftory of the *Rhododendron Chryfanthum*. The medical account is taken chiefly from Koelpin (fee our 1ft vol. p. 226.). This plant which is the *Rhobodendron ponticum* of Linnæus, is defcribed by Tournefort under the name of *Chamærodendros pontica maxima, folio lauro cerafi*, and has been found by Alftroemer, a Swedifh naturalift, in the neighbourhood of Gibraltar. It grows fpontaneoufly in the Levant, on the coafts of the Black Sea, in the Ifles of the Archipelago and Kamfchatka, and along the Amur in Ruffia, Siberia, and China. It abounds moft in rich foils, along rivers and lakes, and in the fhade.

---

An Academy of Sciences has lately been inftituted at Lifbon. It is compofed of Twenty-four ordinary and the fame number of Honorary Members. Twelve of the latter are to be foreigners. There are likewife fupernumerary and correfponding Members. The Queen of Portugal has given the Academy apartments in the

Palace

Palace *das Neceſſidades*, and the excluſive privilege of printing almanacks.

---

A third volume of Dr. Cullen's *Firſt lines of the practice of Phyſic* is expected ſoon to be publiſhed at Edinburgh.

